# A qualitative study to identify parents’ perceptions of and barriers to asthma management in children from South Asian and White British families

**DOI:** 10.1186/s12890-017-0464-9

**Published:** 2017-09-20

**Authors:** Monica Lakhanpaul, Lorraine Culley, Noelle Robertson, Deborah Bird, Nicky Hudson, Narynder Johal, Melanie McFeeters, Emma Angell, Charlotte Hamlyn-Williams, Nadine Abbas, Logan Manikam, Mark Johnson

**Affiliations:** 1 Population, Policy and Practice, UCL Great Ormond Street Institute of Child Health, 30 Guildford Street, London, WC1N 1EH UK; 2 School of Applied Social Sciences, De Montfort University, The Gateway, Leicester, LE1 9BH UK; 3 Clinical Psychology, University of Leicester, Centre for Medicine, Lancaster Road, Leicester, LE1 7HA UK; 4 Department of Community Paediatrics, Buckinghamshire Healthcare NHS Trust, Wycombe Hospital, Queen Alexandra Road, High Wycombe, HP11 2TT UK; 5 Parent representative, Leicester, UK; 6 Specialised Commissioning East Midlands, NHS England, Fosse House, 6 Smith Way, Grove Park, Leicestershire, Enderby LE19 1SX UK; 7 Department of Health Sciences, University of Leicester, University Road, Leicester, LE1 7RH UK; 8 School of Medicine, University of Southampton, University Road, Southampton, SO17 1BJ England; 9 Faculty of Health and Social Studies, Mary Seacole Research Centre, De Montfort University, The Gateway, Leicester, LE1 9BH UK

**Keywords:** Asthma management, Children, South Asian, Qualitative, Barriers, Participatory, Tailored, Interventions

## Abstract

**Background:**

Over one million children receive treatment for asthma in the UK. South Asian children experience excess morbidity and higher rates of hospitalization than the White population. This study aimed to explore perceptions and experiences of asthma and asthma management in British South Asian and White British families, to identify barriers to optimal management and to inform culturally appropriate interventions to improve management.

**Methods:**

A qualitative methodology, using semi-structured interviews was adopted. Members of 30 families from six major South Asian ethnic-religious groups were purposively sampled (*n* = 49). For comparison, 17 White British parents were interviewed. Topics included understandings of asthma; day-to-day management; interactions with health care providers and the perceived quality of healthcare services. Data were analyzed using interpretive thematic analysis, facilitated by NVivo. Similarities and differences between South Asian and White families were analysed across key themes.

**Results:**

Many of the problems facing families of a child with asthma were common to South Asian and White British families. Both had limited understanding of asthma causes and triggers and expressed confusion about the use of medications. Both groups reported delays in receiving a clear diagnosis and many experienced what was perceived as uncoordinated care and inconsistent advice from health professionals. No family had received an asthma plan. South Asian families had more difficulty in recognising severity of symptoms and those with limited English faced additional barriers to receiving adequate information and advice about management due to poor communication support systems. South Asian parents reported higher levels of involvement of wider family and higher levels of stigma. Attendance at the emergency department was related to previous experience, difficulties in accessing primary care, lack of knowledge of alternatives and difficulties in assessing severity.

**Conclusions:**

Barriers to optimal asthma management exist at the individual family, community and healthcare systems levels. Culturally sensitive, holistic and collaboratively designed interventions are needed. Improved communication support for families with lower proficiency in English is required. Healthcare professionals need to ensure that families receive an asthma plan and make greater efforts to check families’ understandings of asthma triggers, use of medications, assessment of asthma severity and accessing help.

## Background

Recent reports have shown that the outcomes for children in the UK with chronic disease are amongst the worst in Europe. Death rates for illnesses that rely heavily on primary care services such as asthma, are higher in the UK in comparison to other European countries [1, 2]. There is evidence to suggest that UK children’s health services provide inferior care compared to equivalent European countries [3].

Asthma is a common childhood chronic condition. Estimates suggest that 1.1 million children, or 1 in 11 children in the UK, will experience asthma at some point in their childhood [4]. Every year 44,000 children are admitted to hospital due to asthma, with 40 to 50 children dying, on average, as a result of their asthma during admission [4]. The number of reported asthma deaths for 5- to 34-year olds in the UK is one of the highest in Europe [5].

Evidence also indicates that 75% of hospital admissions for children with asthma could have been prevented with better primary care [1]. The NHS spends around £1 billion per year on asthma management [4, 6]. The management of asthma in children is costly, related to investigations prior to diagnosis, acute and primary care visits and asthma medication [7]. Additionally, the cost of a visit to the Emergency Department (ED) is approximately £132 per patient [8]. Further costs are attributed to subsequent investigations: if a referral results in an admission to a ward, the cost can comprise as much as £1000 for each 24-h period [7]. There are also significant social implications for child and family, notably time lost from school, adverse impact on educational attainment and emotional well-being. Working parents or carers also require time off work for childcare due to the consequences of suboptimal management of asthma [9].

Managing asthma requires long-term follow up by health care professionals (HCPs). However, current evidence demonstrates that some minority ethnic groups experience inequalities in their health care [10, 11]. Minority ethnic groups often experience higher morbidity and mortality, and poor outcomes in comparison to the majority populations for a range of chronic diseases [10, 11]. The need to find effective interventions to address these health inequalities in outcomes is, therefore important, and widely recognised both by governments [12, 13] and physician groups [14].

In the UK, individuals of South Asian origin with asthma experience excess morbidity, with hospitalisation rates three times higher than those of White British origin [15]. Children in South Asian communities in the UK are known to suffer from under-recognised asthma symptoms and disease severity, and are more likely to experience uncontrolled symptoms and be admitted to hospital with acute asthma compared to White British children [15–17] despite no evidence of greater asthma severity. For example, in Leicester, the hospital admission rate and ED attendance for South Asian children is approximately 4 times higher than White British children [17]. Further data derived from the UK indicate that South Asian children with asthma are more likely to suffer uncontrolled symptoms and be admitted to hospital with acute asthma in comparison to White British children. The inequality needs to be addressed since inadequate asthma management increases asthma morbidity, service utilisation [18, 19] and affects quality of life [20].

Barriers to asthma management in South Asian children are well documented, and many health promotion interventions aimed at improving asthma outcomes are unsuccessful in South Asian children because they lack cultural sensitivity [21, 22]. Indeed there are often poor health outcomes within the UK because services are planned around the needs of organisations and service providers, rather than the children and families [3]. A systematic review identifying key barriers to optimal management of asthma in South Asian children [21] noted numerous barriers to be culturally driven including; diverse beliefs regarding the nature of asthma, under-use of preventer medications (due to fears of over-use), and impact of prejudice and stigmatisation. Additional key issues were reliance on EDs in preference to primary care and difficulties in perception of symptom severity. Service level issues such as poor communication and support were also contributing factors [21, 22]. Such findings suggest a need to work more closely with communities to understand parents’ and carers’ perceptions of the barriers to optimum management and to determine families’ perceptions of how services can be improved.

The challenge for researchers, clinicians and policy makers is to identify the variations in care and their cause, and to explore how they can be addressed through service improvements for all users and the development of tailored, culturally sensitive interventions to address specific needs of minority ethnic groups.

This paper is drawn from the Management and Interventions for Asthma project (MIA) [9]. The aim of this study was to explore perceptions of asthma amongst South Asian families using a socio-ecological approach to identify key issues that prevent optimal management, ensuring that they can be addressed in future interventions. A comparative White British sample was used to identify if the management of asthma was subject to variation between communities in a single local area, so that generic themes related to all children and those requiring tailoring for religion or culture could be identified. In addition to the data reported here, the study included a community-based study, including focus groups and interviews with key informants and with healthcare professionals and the development of a framework for intervention planning [9].

Working with individuals and communities to define problems and develop solutions is one of the fundamental principles of participatory research and a guiding principle of the MIA project [9]. Participatory research does not replace other forms of research, but instead defines how the research process is carried out. Most interventions to improve asthma care are directed at majority populations with few having been developed using a participatory approach. The study aimed to address both issues: tailoring for the needs of the South Asian community and also developing a participatory staged approach that could be translated to other communities and other clinical areas.

This was complemented by the use of a socio-ecological model of health and intervention design. Ecological models provide comprehensive frameworks for understanding multiple and interacting determinants of health behaviours and can be used to develop comprehensive intervention approaches that systematically target each level of influence [23].

## Methods

The study adopted a qualitative methodology, using semi-structured interviews with parents, and carers.

### Sampling frame

Purposive sampling was used to ensure proportional representation from the six main South Asian ethno-religious groups (Indian Gujarati Hindu; Indian Gujarati Muslim; Pakistani Muslim; Bangladeshi Muslim; Indian Punjabi Sikh and Indian Punjabi Hindu), with participants being asked to self-assign their ethnicity. Purposive sampling was also used to identify children with a wide range of asthma severity within each ethnic group. Severity was defined according to the British Thoracic Society (BTS) guidelines ‘stepwise approach to management’, which places a child’s asthma control in one of five steps or categories of asthma [24]. The aim was to sample sufficient families to ensure that a range of South Asian ethnicities and range of asthma severities were encompassed by the research. When data saturation was reached, no further families were recruited. Purposive sampling was subsequently used to select a sample of White British families that broadly matched the South Asian families. Only families that had children aged 4–12 years with diagnosed asthma were included.

### Recruitment

Multiple recruitment avenues were used to identify and approach families in order to ensure variability of experience within the sample. Table 1 identifies the numbers of families approached via the various recruitment avenues.

**Table 1:** Recruitment avenues and numbers for the South Asian families

Recruitment numbers	Recruitment methods											
	Direct approach in the community				Letters		Telephone	Opportunistic recruitment				
	Advertising	By CFs	By the research team	Community events	Mail shot	Letters to recent attendees	GPs	GPs	Pharmacies	A&E	Asthma clinic	Paediatric clinics
Number of Expression of interest forms returned	0	11	7	7	0	1	10	6	21	7	3	8
Ineligible	0	0	1	1	0	0	2	0	4	0	0	0
Not recruited	0	1	2	1	0	0	5	5	11	2	2	6
Recruited	0	10	4	5	0	1	3	1	6	5	1	2

### Data collection

Interviews explored the following topics: understandings of asthma; family and community perceptions of asthma; day to day management; medical management; interactions with health care providers and the quality and improvement of healthcare services and provision. Demographic information was collected, with families given the choice whether they wished to be interviewed together or as individuals. Interviews took place in the families’ homes, were digitally recorded and transcribed, and translated into English where required by community facilitators specially trained by the research team to assist in data interpretation [9].

### Data analysis

The South Asian and White British family interviews were analysed according to the principles of interpretive thematic analysis and facilitated by the use of NVivo [25].

The analytic process for the family interviews began with coding of the South Asian dataset using a process of open coding, followed by the development of emergent themes and the clustering of themes in an interpretive process. The basic codes were then elaborated into a framework of thematic categories within NVivo. All South Asian family interview transcripts were then coded using the resultant framework.

The White British interviews were subject to an independent process of open coding by a second analyst in order to avoid undue influence from the existing coding framework developed from the South Asian family interviews. The emergent White British codes were then closely compared to the existing South Asian coding framework. Where existing codes were conceptually transferable, these were preserved in the framework. A small number of nodes were added that were not present in the South Asian families’ data. Similarly, some nodes remained in use for the South Asian data but were not used in analysing the White British data as no instances were found. Subsequently, some of the generic nodes were discussed by both analysts and were amended to ensure consistency of meaning and validity across both data sets.

The existing framework was amended to allow applicability to the entire data set. This became the final, comprehensive coding framework. The South Asian data were subsequently revisited by the first analyst at this stage, to ensure that integrity of the coding was maintained following the changes. All transcripts were systematically coded in NVivo using the resultant comprehensive framework. This allowed further case and cross-case analysis to be performed between and within sub-groups [25].

### Ethics

Ethical approval was gained through the local NHS ethics committee, University ethics and NHS R&D approval. Health Research Authority procedures were used throughout the study to minimise distress to participants, safeguard anonymity and confidentiality, as well as providing a guarantee for a continued standard of care [9].

## Results

### Participants

Thirty South Asian families participated, consisting of 44 parents (29 mothers, 15 fathers), five secondary carers (4 females, 1 male) with a total of 49 South Asian interviewees (Table 2).

**Table 2:** Demographic information of South Asian participants^a^

	Indian Gujarati			Pakistani	Bangladeshi	Indian Punjabi		Total
Religion	Hindu	Muslim	Jain	Muslim	Muslim	Sikh	Hindu −^a^	
Mothers	6	4	1	4	7	6	1	29
Fathers	4	3	1	2	1	3	1	15
Secondary Carers	0	0	0	1	1	3	0	5
Total	10	7	2	7	9	12	2	49

^a^Leicestershire has a significant Jain population – a sub-group of the Hindu faith

Children were aged between 5 and 12 years old: seven at BTS level 1, 17 at BTS level 2, six at BTS level 3, and three at BTS level 4.

Fourteen White British families were recruited including: 17 parents (13 mothers, 4 fathers). Children were aged between 5 and 11 years old, three at BTS level 1, eight at BTS level 2, and three at BTS level 3.

### Themes

The key themes arising from exploration of the parents’ and carers’ data are described below: Beliefs and understanding of asthma; medicines; non-medical management; control of asthma; interacting with NHS services and impact of asthma (Table 3).

### Beliefs and understandings of asthma

When speaking about beliefs and understandings about asthma, families focused on aetiology and exacerbation, and previous knowledge and experience of asthma.

#### Causes of asthma

South Asian and White British families discussed their understanding about the causes or origins of asthma. ‘Causes’ of asthma and exacerbations or ‘triggers’ for asthma were often confused in the interviews in both groups. The most commonly mentioned causes across all families were environmental (e.g. the weather, damp, dust, pollen, pollution), and physiological and genetic (e.g. weak immune system, asthma being hereditary, not breast feeding). Nine (30%) South Asian families and five of 14 (36%) White British families felt that the cause of asthma was either not known or not identified.

*“…there’s no sort of identifiable cause but it is very common, becoming more common”. (Mother, Indian Gujarati)*

Eighteen (60%) of South Asian families discussed religion or fate, discussing either God’s will (Muslim participants) or Karma (Hindu participants), i.e. that their child’s asthma was destined to be, as the cause of their child’s asthma. No White British families discussed fate or religious beliefs.

#### Reaction from others

South Asian families discussed the types of reactions they received when they had informed family members or others within their community about their child’s asthma. A number of negative reactions were cited, including fear that asthma was ‘contagious’ or was caused by ineffective parenting. A small number of South Asian mothers felt blamed for their child’s asthma. None of the White British families reported such negative reactions from others, though one mother blamed herself due to smoking during pregnancy.

*“It might make a change because they blame me. [My extended family] just say, tell me that I don’t take care of her. That’s what they say to me. [Laughter] I’m not taking care of her.*.  *. that’s what they think and then I just turn around and I say, what makes you think that? Is it because I’m working? Because I enjoy working? I’m not going to stop working because she’s ill, because that ain’t going to make her better. If I stop working, I can’t sit down with her twenty-four seven and say, Oh you feeling better? I can’t keep her from school and say Oh, don’t go to school, you’re going to get ill.” (Mother, Bangladeshi).*

#### Information about asthma

Both South Asian and White British families reported a lack of information provision by health care professionals about their child’s asthma. Specifically, there was a lack of written information about asthma management. No family in the study reported having received a written asthma plan. Many families (8 (57%) White British families and 19 (63%) South Asian families) reported wanting more information about asthma and its management.

A number of South Asian families reported being unable to communicate directly in English which greatly hindered expression of preferences. However, in these cases, families had addressed the potential problem by seeking access to HCPs (especially General Practitioners (GPs)) who spoke the same language as they used.

### Medicines

Families prioritised discussion about medicine; in terms of understanding medicines, taking and managing medicines, decisions about medicines, and side effects.

#### Understanding medicines

In both South Asian and White British families there was, or had in the past been, confusion related to the use of inhalers, in particular their function and when to administer them. According to families, this was, in part, due to an inconsistency of information and lack of written information from health care professionals, as discussed above.

*“Because, when the nurse at the hospital said, ‘Has she got an inhaler?’ And I said, ‘A blue one, we’ve been told twice a day,’ she didn’t say, ‘Oh okay, well, actually you can use it up to ten times, and you might find that might more be helpful.’ It was ‘Oh for goodness’ sake, that’s, that’s just a waste of time, it’s a waste of medication, that is.’ You know, and that made me feel like ‘Oh great, so the doctor’s wasted my time and because of that, I’m here.’…I don’t, well, we don’t know. So that would have been helpful. How we control it, and just, just consistent information about how often we should be using the blue inhaler.”. (Father, Indian Punjabi).*

#### Managing administered medication

Most families reported difficulties with administering asthma medicines to children or remembering to take medicines, especially with younger children or children with learning difficulties. In general, in both White British and South Asian families, a parent took responsibility for the medicines until a child was considered old enough to take on this role, often coinciding with starting secondary school and managing their own inhaler use during the school day. A number of families described being unsure about whether their inhaler technique was correct. This was something about which they would have liked more advice.

*“I don’t know if we are still using it right. When he is doing his inhalers he is getting smoke coming out and I am saying to him you can’t be doing it right because that shouldn’t be happening”. (Mother, White British).*

Families gave a number of reasons for why they chose either to adhere to their medication or to increase or decrease their use of medicines. Three (21%) White British families described using the medicines as prescribed, for fear of the consequences for their children’s health if they did not. Reasons for increasing the dosage mainly centered on instances of viruses and colds. Whilst families from both groups described making modifications to their child’s treatment regimen, South Asian families more often discussed *not* giving medicines or reducing the dosage because of concerns about side effects or over-dependence. Other common reasons for not adhering to medicines included a lack of clear diagnosis, an absence of symptoms at certain times of the year, children refusing or deciding not to take their medicine, and misunderstandings about how to take the medicine.

#### Side effects

Many families (22 (73%) South Asian and 11 (79%) White British) discussed possible side effects deriving from asthma medication, but the South Asian families articulated a wider range of concerns. These included children’s growth, stomach problems, heart problems, addiction, reduced immunity, oral thrush, mood or behavioural issues, and reduced immunity due to drugs. One South Asian parent believed that medicines made their child’s asthma worse. White British families talked mainly of general concerns about long-term use of medicines, specifically mentioning effects of steroids on weight, fears of overdose, behavioural problems, bowel problems and dry mouth/cold sores. Concerns about long term use of steroids, particularly in relation to dependence caused by ‘overreliance’, was a concern for both South Asian and White British families. It affected the decisions families made about using medications.

*“That stuff can actually, sort of [affect his] growth or his bones can be a bit thinner bones. We were more sort of thinking, that if he’s going to have steroids, we were not worried about the pumps, giving steroids the pink tablets, which we were concerned about, because he was given that, let’s say, every 4 weekly, every 3 weekly.*.  *. Because his body, immune system, would not work if you keep feeding him steroids and his immunity will be so broken down he’ll have to rely on steroids for the rest of his life, which I wouldn’t want.” (Father, Indian Gujarati).*

### Non-medical management of asthma

Both South Asian and White British families described using non-medical management to try to help control the symptoms of their child’s asthma. Non-medical management included making adaptations in the home, as well as dietary modifications, religious solutions, and non-asthma medications. Changes in the home included damp dusting, vacuuming, changing beds more frequently, improving ventilation, changing flooring and not having pets. South Asian families described taking extra direct measures involving the child, including keeping them warm or indoors, and rubbing and massaging or patting the back.

Whilst diet was mentioned by White British families (including discussions around the importance of healthy eating and cutting down on junk food), South Asian families more often spoke of altering their child’s diet in an attempt to control symptoms; all South Asian families alluded to this practice. Avoiding physically cold foods such as ice-cream and giving warm foods was a common strategy, as was the use of herbal supplements such as turmeric.

### Control of asthma

Both South Asian and White British families talked about keeping their child’s asthma under control. However, the perception of asthma control varied amongst participants. There was some confusion whether ‘asthma control’ related to formal measures of asthma control used by their health care providers or the non-medical management behaviours carried out in order to keep things ‘under control’.

*“I don’t want to give him puffs all the time when he doesn’t need it. So I’m rather hopeful as he grows up, he grows out of it so as long as he is getting better and you are giving him when he needs it and as he gets old he doesn’t have this problem to just try and keep it in control.” (Mother, Pakistani).*

However, a number of parents related asthma control to the regular and appropriate use of asthma medications.

*“I think a lot of it is to do with the medication, if he came off his medication I think he would be quite a poorly child”. (Father, White British).*

Some families also reported that after trial and error and tailoring of medication they were now more able to achieve good control of their child’s asthma.

### Interacting with NHS services

Interview data demonstrated that for families who had a child with asthma, there were three key ‘moments’ when they might interact with the NHS. These were: (1) during the process of getting a diagnosis (mostly with the GP but in some cases with ED staff); (2) when the child had an acute asthma attack (NHS Direct, Out of Hours service, ED); and (3) during on-going management (most likely to be the practice nurse, or for some South Asian families, the pharmacist).

#### Diagnosis

Both South Asian and White British families reported difficulties with getting a diagnosis. Seven (50%) White British families and 13 (43%) South Asian families reported being prescribed asthma medication even though they had not initially been given a diagnosis. South Asian and White British families described feelings of not being taken seriously. Multiple visits to the GP with recurring problems and delays in receiving a diagnosis engendered feelings of annoyance and anger. The feeling that HCPs were reluctant to make a diagnosis led to confusion, as families did not understand why asthma medication was given without a diagnosis.

“It’s just getting people to listen, that’s what used to get me so angry because nobody would listen to me. When I used to take him doctors I used to mention my nephew had the same symptoms and now he is on all these inhalers. ‘Because your nephew has got these symptoms don’t mean to say you have got the same thing, it’s just chest infection, it’s just something it’s just common to him’, that’s what I used to get. I used to go in there and come out upset all the time”. (Mother, White British)

Families’ felt the absence of a diagnosis for their child’s symptoms created uncertainty about whether to administer medicines and how to manage symptoms. A number of families reported relief on receiving a diagnosis, feeling that this conferred a status that gave them access to asthma reviews and medications, and facilitated their communication about the condition to their child and to others, notably the child’s school.

#### Acute attacks

A significant proportion of families had required NHS access in an emergency or non-routine situation, requiring decisions from the parents concerning (1) the severity or (un)usualness of the child’s symptoms; and (2) where to seek help. Both South Asian and White British families perceived the GP to be the first point of contact in managing their child’s asthma. However, when symptoms were perceived to be beyond the expertise of their GP, or if the practice was closed (during evenings and weekends), White British families were more likely to use their own doctor’s out-of-hours service (nine (64%) White British families compared with three (10%) South Asian families) rather than other options, such as walk-in centres or NHS Direct. In contrast, South Asian families were more likely to self-refer to the ED in an emergency (13 (43%) of families).

*“I just went to my GP and say I’m seeing the same symptoms. They referred him to the hospital. And [we’ve] just been going from there”. (Mother, Pakistani).*

A higher proportion of White British families expressed a belief that the GP was not an expert in asthma and, as a consequence a small number of families felt that they should have been referred to an asthma ‘specialist’. However, when South Asian families felt dissatisfaction with their GP, they were more likely to attend the ED. Some South Asian and White British families had received information relating to an asthma diagnosis via the ED HCPs and, in some cases, this was interpreted as meaning that the staff at the hospital had superior expertise and knowledge about asthma. Parental knowledge about alternatives to presentation at ED appeared limited.

#### Ongoing management

The ongoing management of a child’s asthma often required that families interacted with HCPs regularly, usually through a regular or annual asthma review with a practice nurse. Most families who described attending asthma reviews (approximately a third of South Asian families and two thirds of White British families) were satisfied with this process. Here, the asthma review was seen as a process that involved checking things like inhaler technique and peak flow rather than receiving an asthma diagnosis, receiving new information or the review of asthma medication.

*“It’s more a question and answer session as to how they have been, but that’s not a review of their medication because it’s the doctor surely who has to sanction any new or alterations.” (Father, White British).*

The nurses who provided this service were viewed positively by both South Asian and White British families. In the families not accessing an annual review (more likely to be South Asian families), this often appeared a result of either devaluing the review or because they were seeking advice from other sources, such as pharmacists. Almost a third of South Asian families described accessing the pharmacist for advice about a child’s asthma and medications; to receive a second opinion, to gain additional information about asthma medicines, and, in some cases, the pharmacist demonstrated how to use inhalers or spacers. For parents who had difficulties with English, using a local pharmacist who spoke the same language consolidated and clarified the information given to them by the GP.

Only three (21%) White British families discussed attending a pharmacist, all three describing trying to access medicines in an emergency without a prescription.

### Impact of asthma

Parents discussed asthma’s impact on them, their families and their children. For parents and carers, dominant impacts centred on taking time off work (four South Asian parents, two White British parents) and the emotional impact on their families, such as the fear and worry caused by seeing their child ill or distressed (23 (77%) South Asian families, five (36%) White British families).

Whilst many families felt that asthma should not dominate their child’s life, six (20%) South Asian families and two (14%) White British families talked about children missing activities or sport because of their asthma. For some South Asian families there was an additional concern and worry regarding what a diagnosis of asthma may mean for their child as they grew older, including potential effects on future job prospects, and, for a small number of families with a daughter, consequences for future marriage prospects. No such concerns were expressed by British families.

*“It’s quite shock...shocking really. I’m quite traumatised at times because it was.*.  *. a couple of times we had to take her in and out, it wasn’t just once. It was like coming.*.  *. straight after she was diagnosed it was so difficult and, on occasion, every 2–3 months, this was a couple of times we went to the hospital. The first thing it was go to A&E and I don’t think you’re allowed to go to A&E now for such things unless it’s really, really severe. Yeah, all we did for five hours in hospital or even longer, half the night in hospital we had to spend with her while she was on the bed and she’ crying and screaming and they put the nebulisers on.*.  *. and it’s quite.*.  *. she’s so small and tiny. Tiny little girl. Poor thing. She was screaming, she didn’t like the mask thing on her face, it was happening all the time and she was getting scared of hospitals, was thinking ‘what was going on?” (Mother, Bangladeshi).*

### Summary of similarites and differences between South Asian and White British families

**Table 3:** Summary of similarities and differences between South Asian and White British families across themes

Similarities	Differences
Beliefs and Understandings of Asthma	
Environmental and physiological/genetic factors were the most commonly mentioned ‘causes’ across all groups	Fate, destiny or religion were more likely to be mentioned by South Asian families
Approximately one-third of all families specifically stated that they felt unclear about the origins of asthma	South Asian families were more likely to mention the UK environment as a trigger of an asthma exacerbation
The perception that asthma is something children can grow out of was similar across both groups	South Asian families were less likely to have prior knowledge about asthma unless they had familial experience of it
	White British families were more likely to know that asthma was a condition that existed even without previous familial experience of it
Reactions from others	
Extended families, friends and wider community often offered advice about asthma management. Families often did not consider this an important or reliable source of information about asthma	South Asian families were more likely to report receiving advice from extended family, especially in relation to alternative therapies and remedies for asthma. Advice sometimes extended to family members living in South Asia
	South Asian families were more likely to have extended family living with them and these relatives were more likely to have a role in caring for a child with asthma
	South Asian families were more likely to report negative reactions from others about their child’s asthma
Information about asthma	
A lack of timely, consistent information-giving by HCPs was reported by both South Asian and White British families	South Asian families whose first language was not English reported needing to access primary care staff who spoke the same language
Families would prefer information to be given both face to face by HCPs and in written form for later use	
Consistency of advice and information from all HCPs was important for both South Asian and White British families	
No one had received a written asthma plan	
Medicines	
Both South Asian and White British families were often confused about the correct use of medicines for asthma and in some cases about inhaler technique	South Asian families mentioned a wider range of possible side effects
Parents generally took responsibility for medicines until a child was considered able to do this for him- or herself	
Families used a number of strategies to help with adherence, including the use of spacers	
South Asian and White British families had concerns about the side effects of asthma medicines, particularly in relation to the long-term use of steroids	
Both South Asian and White British parents described actively making decisions to increase or reduce the dosage of medicines given to children, which was sometimes at odds with the advice they had received from HCPs	
Non–Medical Management	
Both South Asian and White British families discussed the adaptations and non-medical management strategies they have tried to relieve asthma symptoms	South Asian families tended to use more additional measures such as keeping the child warm
Both groups had made changes to the home and environment	South Asian families were more likely to try religious, herbal or alternative therapies and modify a child’s diet
Interactions with the NHS	
Getting a diagnosis for asthma was experienced often as a difficult and lengthy process for both South Asian and White British families	White British families were more likely to describe accessing the GP out-of-hours service in an emergency or non-routine situation
There was a perception among families that HCPs (usually the GP) did not take their concerns seriously or were reluctant to make a diagnosis	South Asian families were more likely to describe self-referral to the ED
Not having a diagnosis for their child’s symptoms led to a great deal of uncertainty about the best course of action for families	South Asian families were less likely to be attending an asthma review and were more likely to be seeking additional information from pharmacists
South Asian and White British families had similar concerns about the quality of healthcare services and staff. These particularly related to inconsistency, HCPs not doing their job properly and ineffective communication skills	Concerns over the quality of health care were exacerbated for those whose first language was not English
South Asian and White British families valued staff who were knowledgeable, informative and able to communicate effectively, who were supportive, caring and were child-friendly	

## Discussion

This study offered an opportunity for parents and carers to discuss their experiences of asthma care and perceptions of barriers to optimal asthma management. The study has shown the multifaceted nature of barriers to optimum management, which reside at the family, community and healthcare system level and thus strategies to address change need to take into account a range of potential influences on care. The Children’s Outcome Forum report recommends that when considering strategies for improving outcomes for children, we should work in close partnership with families, children and young people [1]. The detailed interviews with parents also revealed that many of the problems facing families of a child with asthma are common to all communities. However, the findings also revealed several key issues appearing especially pertinent to South Asian families, highlighting the need for a tailored approach when designing interventions to improve the management of asthma in South Asian children.

The findings indicated that understanding asthma, including the recognition of symptoms, triggers and management strategies, is central to effective asthma management for children in all ethnic populations. Having this knowledge supports the development of a partnership between families and health professionals and increase the opportunity for the child and family to be fully involved in decision-making.

In our study, both South Asian and White British families’ highlighted issues related to the consistency, timeliness and quality of the information provided regarding their child’s asthma which, when sub-optimal, was linked to confusion about different medications available and the techniques required to deliver them (e.g. how to use inhalers). This appeared particularly problematic for those with limited English, in the absence of appropriate communication support systems. It is important that families are provided with information using multiple delivery mechanisms. This should not only include face-to-face and written information but also audio and visual formats to address language and literacy issues. These formats will also allow for information to be shared with the wider family.

This study revealed that all families, irrespective of ethnicity, encountered barriers related to information provision or misconceptions regarding causes, triggers and the nature of asthma. However, these barriers seemed more pronounced for South Asian families, associated with language or translation issues. Where families had no previous exposure to asthma prior to diagnosis, South Asian families tended to be less familiar with the characteristics and management of the condition compared to White British families. Healthcare professionals should not assume prior understanding of asthma and understanding should be established with families at the beginning of any consultation. Interestingly, the South Asian families did not directly raise language issues as a dominant barrier. It may be that in seeking out GPs or pharmacists speaking the same language or attending ED, where interpreters were more easily available, they developed the means to address such challenges.

This could also offer an important insight into the reasons for the higher usage of these particular services by specific populations due to easier access to interpreters or otherwise language-competent advice, and the changes that need to be implemented to change the pattern of healthcare usage.

South Asian families more often described advice sought and received from the wider family and community, including elders and religious leaders, as well as recourse to complementary therapies, issues often overlooked by healthcare professionals [26]. Healthcare professionals need to clarify what other advice is being sought and shared within the informal, valued communities to better understand influences on asthma management. It is also important to inform such key individuals how to utilise HCPs to ensure the best management for their child and the children in their communities. Asthma education schemes in the UK, such as nurse-led asthma reviews, traditionally focus on the direct family unit of parents (often the mother) and child, with little acknowledgement of the need to include the wider community. An annual asthma review could be adjusted to encompass a wider frame of reference. Community engagement activities to develop awareness and understanding of asthma would also be beneficial. The participatory approach of the MIA study included a community study which demonstrated limited understanding of asthma in South Asian communities and a strong desire for more information [9].

### Accessing care

Within the NHS, primary care services are available to provide an initial diagnosis of asthma and regular reviews to optimise management, with out-of-hours and emergency services available for urgent care and acute severe attacks. In practice, however, emergency attendances for all clinical problems are increasing [3] with a higher attendance rate for South Asian children with asthma compared to White British families [17]. This calls for further research to understand drivers for this health-seeking behaviour if families are to be encouraged and supported to manage their asthma and to choose primary care services in preference to emergency care.

South Asian families in this study chose to access the ED when they believed they were unable to access their GP and were likely to call an ambulance whilst White British families more often used a primary care out-of-hours service. These differences were explained as a response to previous experiences of using the health system (e.g. being referred to the ED during previous acute asthma attacks, or experiencing negative or delayed appointments with their GP) as well as difficulties in the recognition of the severity of symptoms. As almost a third of South Asian families accessed pharmacies to gain further information, the use of pharmacists in asthma care could inform future service models. None of the South Asian families and very few of the White British parents had any formal means of assessing their child’s symptoms. No parents had been provided with written asthma plans and no one reported employing any objective measure of severity. Without being able to assess severity, parents are unable to know at what point and where to access health care for early intervention. A consequence was delay in access to treatment. It therefore must be stressed that helping families to decide how and when to utilise HCPs effectively is vital to ensure the best management for their child and this issue should form an intergral part of any educational intervention with families. It is essential that for all children, national standards regarding provision of asthma plans to children and their families with asthma are met. Consideration may be required as to what is the best format for an asthma plan. HCPs’ need to carefully assess parents’ and children's understanding at each consultation. In addition, South Asian families require additional support to help them identify when and how to access appropriate NHS services.

### Suggestions for service improvements

In this study, both White British and South Asian families expressed feelings of dissatisfaction with elements of the healthcare system. A key issue for many families was a delay in getting a diagnosis, which subsequently led to frustration or anger. Families expressed a desire for a more transparent, systematic and consistent diagnostic process, as current difficulties with diagnosis caused stress and anxiety.

Further difficulties were encountered with what was regarded as inconsistent information and advice from different healthcare professionals. In response to questioning, families and children in both groups offered numerous suggestions to enhance provision and quality of service. Parents requested a more coordinated approach across primary, secondary and tertiary care. Families discussed how an advice centre or telephone service would be useful for when questions arose. Families suggested close engagement with schools to educate and inform children, both those with asthma and the wider community, to improve knowledge and alleviate the stigma associated with asthma and inhalers. Families also suggested that presentations or establishing information centres at easily accessible local community venues, such as temples or community centres. Such venues would be appropriate places for doctors, nurses and people with experience of asthma to provide information about triggers of asthma, discussions of medications and demonstrations of inhaler techniques. Families suggested that information about asthma should be available in multiple languages and formats.

### Strengths and limitations

The study team had prior experience of working with community facilitators and recognised their value in supporting access to identified communities. The study engaged community members as partners rather than subjects, involving them in all stages of research, from identifying research questions to recruitment, developing an intervention, interpreting research findings and disseminating results. Community facilitators were, therefore, central to the success of the recruitment and retention of participants within the study. The snowball sampling method of recruitment adopted in the study can lead to the positive selection of a limited subsection of society. It has many benefits, however, including enabling access to groups otherwise reluctant to engage with any forms of social or health research. Interview schedules were also developed in partnership between the research team and community facilitators.

Taking a participatory approach was extremely resource intensive, both financially but also in terms of staff time and staff engagement. A challenge when using participatory research is that the scope of the project can grow, as it is not apparent from the outset what the needs and agendas of the participants might be. Funders need to be aware of this limitation when considering allocation of resources for such approaches.

## Conclusion

This study reinforces the importance of collaboration with service users and other stakeholders for service improvement and the relevance of exploratory qualitative methods of inquiry. The findings revealed that in all ethnic groups studied, a sound understanding of asthma and its management was lacking. For most families this related to what they perceived as a failure of HCPs to provide consistent and clear information and to check understanding. A number of South Asian families were also faced with an additional language barrier in the absence of appropriate communication support systems, resulting in South Asian families coping in different ways to White British families in the aspects of management such as seeking out help from individuals who could speak their language.

Using collaborative, culturally sensitive, participatory methods [9] allowed the research team to access the perceptions, views and experiences of families and to recognise the priorities of service users. The study identified key issues in the management of asthma, which exist at multiple levels of influence on health outcomes at the patient, provider and system levels. It is therefore, important to work with families to develop a tailored multifaceted intervention programme which incorporates both general strategies and aspects of cultural adaptation in order to address these multi-layered barriers to effective asthma management in children.

## Abbreviations

BTS: British Thoracic Society
ED: Emergency Department
GPs: General Practitioners
HCPs: Health Care Professionals
